# Auxin enhances grafting success in *Carya cathayensis* (Chinese hickory)

**DOI:** 10.1007/s00425-017-2824-3

**Published:** 2017-12-06

**Authors:** R. M. Saravana Kumar, Liu Xiao Gao, Hu Wei Yuan, Dong Bin Xu, Zhao Liang, Shen Chen Tao, Wen Bin Guo, Dao Liang Yan, Bing Song Zheng, Johan Edqvist

**Affiliations:** 1 State Key Laboratory for Subtropical Silviculture, Zhejiang A and F University, Linan, 311300 Zhejiang China; 2 Laboratory of Fruit Quality Biology, Zhejiang Provincial Key Laboratory of Horticultural Plant Integrative Biology, Zhejiang University, Zinjingang campus, Hangzhou, 310058 China; 3 IFM, Linköping University, 581 83 Linköping, Sweden

**Keywords:** ABC transporter, LAX, Nut, Phloem, PIN, Polar auxin transport, Xylem

## Abstract

**Application of auxin to root stock and scion increases the success rate of grafting in Chinese hickory.**

The nuts of the Chinese hickory (*Carya cathayensis*) tree are considered both delicious and healthy. The popularity and high demand result is that the hickory nuts are of very high economical value for horticulture. This is particularly true for the Zhejiang province in eastern China where this tree is widely cultivated. However, there are several difficulties surrounding the hickory cultivation, such as for example long vegetative growth, tall trees, labour-intensive nut picking, and slow variety improvements. These complications form a great bottleneck in the expansion of the hickory industry. The development of an efficient grafting procedure could surpass at least some of these problems. In this study, we demonstrate that application of the auxin indole-3-acetic acid promotes the grafting process in hickory, whereas application of the auxin transport inhibitor 1-*N*-naphthylphthalamic acid inhibits the grafting process. Furthermore, we have identified hickory genes in the *PIN*, *ABCB,* and *AUX/LAX*-families known to encode influx and efflux carriers in the polar transport of auxin. We show that increased expression of several of these genes, such as *CcPIN1b* and *CcLAX3*, is correlating with successful grafting.

**Electronic supplementary material:**

The online version of this article (10.1007/s00425-017-2824-3) contains supplementary material, which is available to authorized users.

## Introduction

The consumption of culinary nuts is often encouraged to reduce the risk of heart disease (Luo et al. 2014; Aune et al. 2016; Musa-Veloso et al. 2016). The positive health effects from nut consumption are probably coming from that many nuts are rich in unsaturated fatty acids and minerals, such as potassium and magnesium (Brufau et al. 2006). The tree *Carya cathayensis* (Chinese hickory) produces culinary nuts, in botanical terminology correctly referred to as drupes or stone fruits, which are considered both highly nutritious and very delicious. This tree is of particular value for farmers in the Zhejiang Province in China where it is broadly planted. However, the problems surrounding the hickory cultivation, such as the long vegetative growth, the tall trees, the labour-intensive nut picking, and the slow variety improvements, form severe obstacles for fast development and expansion of the hickory production. Potentially, the current difficulties could be surpassed through an improved grafting method for hickory. Grafting is an ancient and efficient method employed in various tree species to protect the plants from pathogens, abiotic stresses, and also to increase the yield (Gonçalves et al. 2006; Cantero-Navarro et al. 2016; Corso et al. 2016). Grafting is depending on the development of vascular tissue and reconnection between rootstock and scion (Pina and Errea 2005; Cohen et al. 2007; Melnyk 2017). It is well established that auxin plays a major role in vascular cell differentiation (Fukuda 2004; Woodward and Bartel 2005; Teale et al. 2006).

Nearly 40 years ago, Shimomura and Fujihara (1978) showed that application of the synthetic auxin 1-naphthaleneacetic acid (NAA) to the scion apices stimulated vascular reconnection during grafting of cactus plants. More recently, it was shown in *Arabidopsis thaliana* that auxin accumulation in the grafted area is followed by cell differentiation and vascular reconnection between rootstock and scion (Yin et al. 2012). Furthermore, while auxin induces vascular tissue formation, application of the auxin efflux inhibitor 1-*N*-naphthylphthalamic acid (NPA) reduces the vascular strand formation in the grafted areas of Arabidopsis plants (Wang et al. 2014). In Arabidopsis, auxin and also cytokinin increase at the graft junction. Lowering the auxin level, but not lowering the cytokinin levels, is affecting phloem reconnection, suggesting that auxin has a more important role in the grafting process (Melnyk et al. 2015). In a recent study, overexpression of tryptophan-2-mono oxygenase in root stock resulted in higher auxin levels and improved grafting success rates in tobacco (Li et al. 2017). Hence, auxin seems important for the connection of root stock and scion during grafting.

The polar auxin transport (PAT) allows asymmetric distribution of auxin. This creates auxin gradients, auxin maxima, and auxin minima in tissues. Such variations in auxin levels between neighbouring tissues or cells are extremely important for proper plant development. For instance, PAT has a central role in xylem development in stems and leaves (Růžička et al. 2015). It is expected that PAT is also important for the vein connection during grafting. In Arabidopsis, auxin levels during grafting are peaking in an asymmetric fashion in the pericycle cells adjacent to the xylem (Melnyk et al. 2015). Furthermore, treatment of Arabidopsis cotyledons with the auxin transport inhibitor triiodobenzoic acid (TIBA) suppressed cell proliferation of the vascular tissue during graft union formation (Matsuoka et al. 2016).

Important players in PAT are the family of PIN-FORMED auxin transport proteins (PINs) and the ATP-binding cassette subfamily B (ABCB) proteins (Armengot et al. 2016). Members of both protein families are potential efflux carriers of auxin. There are several studies that suggest an important role for the auxin efflux carriers during regeneration of vascular tissues. For instance, wounding the vascular stems in *Pisum sativum* L. (pea) resulted in increased expression of PIN1, after which the xylem cells differentiated (Sauer et al. 2006). Furthermore, during the regenerative processes after stem wounding in Arabidopsis, there are transient, gradual changes in PIN1 localization. This precedes the complete development of newly formed vascular tissue (Mazur et al. 2016). In *Citrullus lanatus*(watermelon), the expression of several *PIN* and *ABCB* genes is induced during the grafting response (Yu et al. 2017).

The importance of the PIN-proteins during grafting is not undisputed. In Arabidopsis, grafting with PIN mutants did not show altered phloem reconnection compared to wt (Melnyk et al. 2015). However, one should note here that there are eight PIN genes (*PIN1*–*8*) identified in the Arabidopsis genome and there could be functional overlaps between the PIN-proteins (Krecek et al. 2009). In the same study, grafting was not affected by treatment with NPA, which would rather surprisingly indicate a lesser role for PAT in grafting.

PAT is also dependent on the auxin influx carriers. In Arabidopsis, these are the proteins in the AUX/LAX family, AUXIN1 (AUX1), and LIKE AUXIN RESISTANT1–3 (LAX1–3). The *AUX*/*LAX* family encodes multi membrane-spanning transmembrane proteins that share similarities with amino acid transporters (Kramer 2004; Swarup and Péret 2012). Compared to the PIN-proteins, there are less information available concerning the role in grafting for the proteins in the AUX/LAX family. A recent study showed rather surprisingly that most of the *LAX* genes are downregulated during the grafting process in watermelon (Yu et al. 2017). In Arabidopsis, *LAX1* and *LAX3* are upregulated in the presence of auxin, while the expression of *AUX1* and *LAX2* seems to not respond to indole-3-acetic acid (IAA) treatment (Péret et al. 2012). Péret et al. (2012) could also show that *lax2* mutants have vascular breaks in their cotyledons. This developmental phenotype for *lax2* indicates a role for *LAX2* in auxin transport during vascular development. In maize, *ZmLAX1* and *ZmLAX2* are upregulated in shoots and roots in response to IAA treatment, while the expression of three other *LAX* genes in maize remains unchanged (Yue et al. 2015). The expression of *LAX* genes has also been characterized in *Sorghum bicolor* (sorghum) where *SbLAX2* and *SbLAX3* are induced by IAA (Shen et al. 2010).

It is important to improve the grafting methodology in hickory, as this would open up for more efficient breeding and large-scale cultivation. In this study, we aimed to learn more about the role of auxin in the grafting process of hickory. The grafting was followed and evaluated after application of IAA or the auxin efflux inhibitor NPA. Furthermore, hickory genes involved in PAT were identified and their expression patterns during the grafting process under IAA- and NPA-treated conditions were analyzed with reverse transcription quantitative PCR (RT-qPCR).

## Materials and methods

### Plant materials

Cultivation and grafting of Chinese hickory plants were carried out in the Tianmu mountain regions of Zhejiang and Anhui provinces in China. Grafting was done according to the whip and tongue grafting method (described in Lewis and Alexander 2008). The rootstocks used were 2 years old and the scions were 1 year old. For the hormonal application, the tips of the scion and rootstock were dipped in IAA (4 mg/l) or NPA (0.3 μg/l) solution for 10 min, and then, grafting was carried out. Grafting was also performed on scions and root stocks which were not treated with IAA or NPA. These grafts are referred to as the control samples. For each treatment, approximately 30 different clonal samples of rootstock and scion from the graft unions were collected at 0, 3, 7, and 14 days after grafting. After collection, the samples were immediately frozen in liquid nitrogen and stored at − 70 °C for later use.

### Identification and cloning of auxin-transporter genes

The auxin-transporter genes (*PIN*, *ABCB19,* and *AUX/LAX*) were selected from a Chinese hickory cDNA library available to our group (unpublished data). To find out missing 5′ and 3′ ends of the selected genes, rapid amplification of cDNA ends (RACE) was performed with the Clontech RACE kit (Takara, Kyoto, Japan) according to the protocol provided with the kit. Once the full-length gene sequences were identified, the genes were amplified with gene-specific primers (Suppl. Table S1) using the high fidelity DNA polymerase PrimeSTAR Max DNA polymerase (Takara). For the amplification, the reaction was set up in a 50 μl of total reaction volume consisting of 25 μl PrimeSTAR Max DNA polymerase, gene-specific forward and reverse primers (10 μM each) and the respective 5′- or 3′-RACE cDNAs. The amplified fragments corresponding to the estimated gene size were eluted, purified, and cloned into TA cloning vector (pMD18-T; Takara) as described in the manual. The fidelity of the cloned sequence in the plasmid was checked by sequencing at Sangon Biotech (Shanghai, China).

### Sequence analyses

The ORFs of the hickory genes were identified through NCBI ORF finder program (https://www.ncbi.nlm.nih.gov/orffinder). The molecular weight and isoelectric point (pI) for identified protein sequences were calculated using the Expasy online program (http://www.expasy.org/proteomics) (Suppl. Table S2). The transmembrane regions for the hickory PIN and PILS proteins were predicted using TMHMM2 software (http://www.cbs.dtu.dk/services/TMHMM-2.0/) (Krogh et al. 2001). For sequence alignments and phylogenetic analyses, protein sequences were retrieved from Phytozome v12 (https://phytozome.jgi.doe.gov/). Phylogenetic analyses were performed at Phylogeny.fr (http://phylogeny.lirmm.fr/phylo_cgi/index.cgi) (Dereeper et al. 2008). Protein sequences were aligned using Muscle with default settings (Edgar 2004). Alignments were curated with Gblock (Castresana 2000). Phylogenetic trees were constructed using Phyml 3.0 with 100 bootstrap replicates (Guindon et al. 2010). The online tool Interactive Tree Of Life (iTOL) v3 was used for the drawing of cladograms (Letunic and Bork 2016).

### RNA isolation and RT-qPCR

RNA was isolated from rootstock and scion separately at several time points [0, 3, 7 and 14 days after grafting (DAG)] during the grafting process. IAA-, NPA-, and control grafts were used for RNA isolation. Total RNA was isolated using an improved CTAB method where isopropanol was used instead of LiCl for RNA precipitation (Chang et al. 1993). The water dissolved RNA was further purified using the RNA extraction plant aid kid (Sangon Biotech) by following the manufacturers’ instructions. For each time point and treatment, RNA was extracted from triplicate samples. Next, cDNA was synthesized from the RNA samples with the use of Prime Script II Kit (Takara). The reaction setup for cDNA preparation was as follows: 1 μg total RNA, 2.5 μl Oligo(dT) (50 μM), 2.5 μl dNTP mix, and MilliQ H_2_O to adjust final volume to 25 μl. The reaction mix was kept at 65 °C for 5 min and then in ice-bath for 3 min. For the first strand cDNA synthesis, the following compounds was added to the reaction mix: 10 μl 5× prime script RT buffer, 1.25 μl RNase inhibitor, 2.5 μl Prime Script II reverse transcriptase (Takara), and finally, MilliQ H_2_O was used to adjust volume to 50 μl. The reaction mix was kept at 42 °C for 60 min followed by 70 °C for 15 min. The cDNA produced was diluted five times to use in qPCR experiments.

The synthesized cDNAs were used in RT-qPCR experiments. The qPCR analyses were conducted using the CFX96 Real-Time PCR System (Bio-Rad Laboratories, Hercules, CA, USA). For qPCR experiments, primers (Suppl. Table S1) were designed with the Primer3 online software (http://primer3.ut.ee/). The total reaction setup (10 μl) consisted of: 2 μl cDNA, 0.5 μl of each gene-specific primer (10 μM); 5 μl 2 × SYBR qPCR master mix (Takara); 2 μl ddH_2_O. Amplification conditions were as follows: 1 cycle, 95 °C for 1 min; 40 cycles, 95 °C for 15 s, 57 °C for 34 s. After the cycles were completed a read for the dissociation curve was added. For each time point and treatment, triplicate RNA isolations were performed, and each RNA sample was analyzed three times in RT-qPCR. The relative expressions in the samples were calculated by means of 2^−ΔΔCq^ method with 0 day rootstock value as the reference sample. Actin from Chinese hickory was used as a reference gene for the expression analysis (Zheng et al. 2010; Huang et al. 2013; Qiu et al. 2016). The data generated were analyzed by the SPSS 17.0 software (SPSS17 Inc., http://www.spss.com). One-way analysis of variance (ANOVA) was conducted with *P* value of *P* < 0.05 and further statistical significance were interfered with Tukey’s post hoc test (*P* < 0.05).

## Results

### Effect of auxin application on the grafting process

**Fig. 1:**
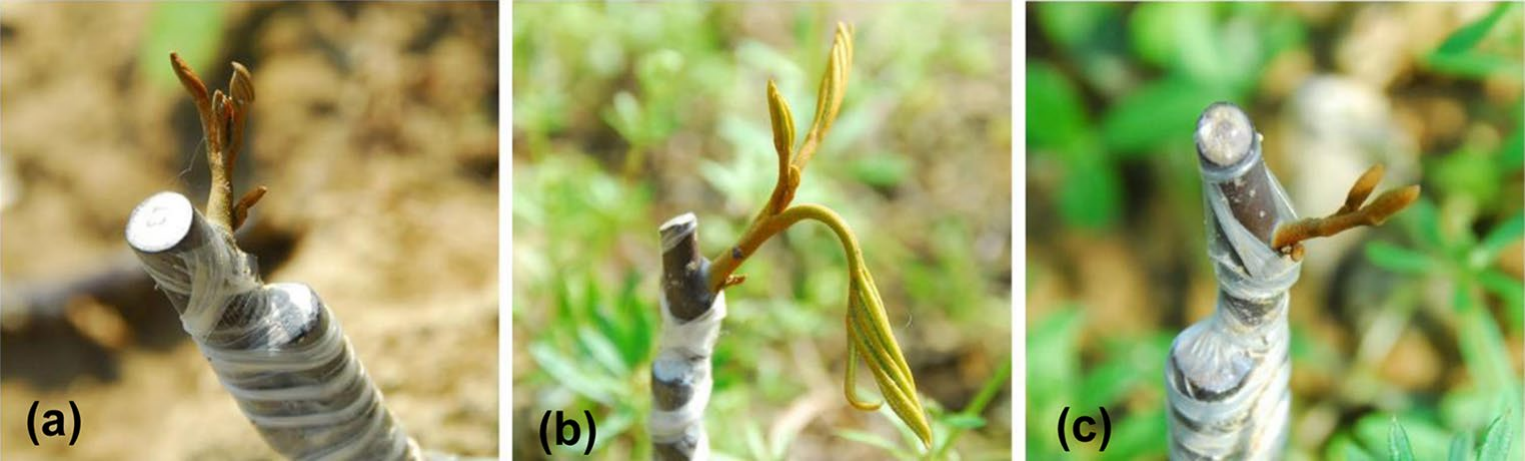
Pictorial representation of the leaf primordial development in the grafted plants in control (**a**), after IAA treatment (**b**), and NPA treatment (**c**) at 30 days after grafting (DAG)

The effect on auxin over the grafting process was studied by applying IAA and NPA to the grafted rootstock and scion samples. The outcome of the grafting was scored at 30 days after grafting (DAG). Plants that survived and showed leaf expansion at 30 DAG were scored as successful graftings (Fig. 1). Overall, the application of IAA improves the grafting success to 80% and NPA application lowers the grafting success to 24%, while the non-applied control plants showed 32% grafting success (Table 1).

**Table 1:** Success rate of grafting under different conditions

Treatment	Plants grafted (no.)	Successful graftings (no.)	Successful graftings (%)
Control	31	10	32
IAA	35	28	80
NPA	29	7	24

### Identification and expression of auxin efflux transporters in hickory

**Fig. 2:**
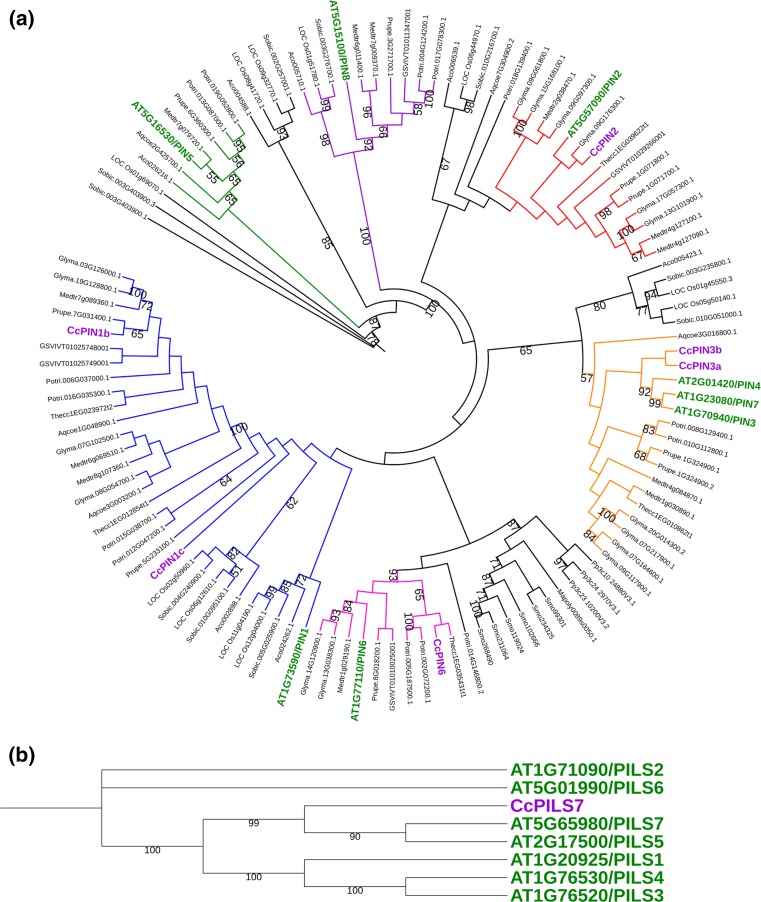
Cladogram including the newly identified hickory CcPIN (**a**) and CcPILS (**b**) proteins. PIN and PILS proteins from different plants such as *Arabidopsis thaliana* (AT), *Vitis vinifera* (grapevine) (GSVIV), *Marchantia polymorpha* (Mapoly), *Physcomitrella patens* (Pp), *Selaginella moellendorfii* (Smo), *Theobroma cacao* (Thecc), *Prunus persica* (Prupe), *Glycine max* (Glyma), *Medicago truncatula* (Medtr), *Populus trichocarpa* (Potri), *Sorghum bicolor* (Sobic), *Ananas comosus* (Aco), *Oryza sativa* (LOC Os), and *Aquilegia coerulea* (Aqcoe) were retrieved from Phytozome v12 (https://phytozome.jgi.doe.gov). Bootstrap values over 50 are shown. For orientation, the names of the hickory proteins are shown in purple, and the Arabidopsis proteins are in green. Clusters corresponding to different PIN-proteins are shown in different colours. The sequence identifiers are from Phytozome v12 (https://phytozome.jgi.doe.gov)

Since auxin seems important for the grafting process, we decided to investigate the expression during grafting of genes involved in PAT. In the Arabidopsis genome, eight *PIN* genes have been identified (*AtPIN1*–*8*) and characterized (Bennett 2015). We used the Arabidopsis *PIN* sequences to search for *PIN* coding cDNAs in a database obtained from the sequencing of a hickory cDNA library. In this way, we could identify six hickory *PIN* genes. Based on the phylogenetic analysis of *PIN* gene sequences from hickory, Arabidopsis, and other plants, the newly identified hickory genes were classified and grouped (Fig. 2). The hickory *PIN* genes were named (*CcPIN1b*, *1c*, *2*, *3a*, *3b*, *6*) according to their phylogenetic clustering with the Arabidopsis *PIN* genes, as described below.

Two of the hickory PIN-proteins located to the same cluster as Arabidopsis PIN1; these two hickory proteins were named CcPIN1b and CcPIN1c. Several *PIN1* genes have also been identified in other plants, such as in *Oryza sativa* (rice) which encode *PIN1a*, *1b*, *1c,* and *1d* (Wang et al. 2009). Two hickory PIN-proteins were clustering together with the Arabidopsis PIN3, PIN4, and PIN7. These hickory PIN-proteins were named CcPIN3a and CcPIN3b. In addition, in other species, for instance in *Glycine max* (soybean) and *Phaseolus vulgaris* (common bean), the *PIN* genes in the *PIN3/PIN4/PIN7* cluster have been named as *PIN3x* rather than *PIN4* or *PIN7* (Wang et al. 2015; Liu and Wei 2017). Hickory *PIN* genes corresponding to the Arabidopsis *PIN5* and *PIN8* genes were not identified. The number of hickory *PIN* genes may increase once the complete hickory genome sequence is available. It is noteworthy that the hickory *PIN* genes always clustered with genes from other woody plants such as *Prunus persica* (peach), *Populus trichocarpa* (poplar), *Theobroma cacao* (cocoa tree), and *Vitis vinifera* (grapevine). We also observed that all PIN-proteins from non-seed plants formed a separate clade in the phylogenetic tree.

**Fig. 3:**
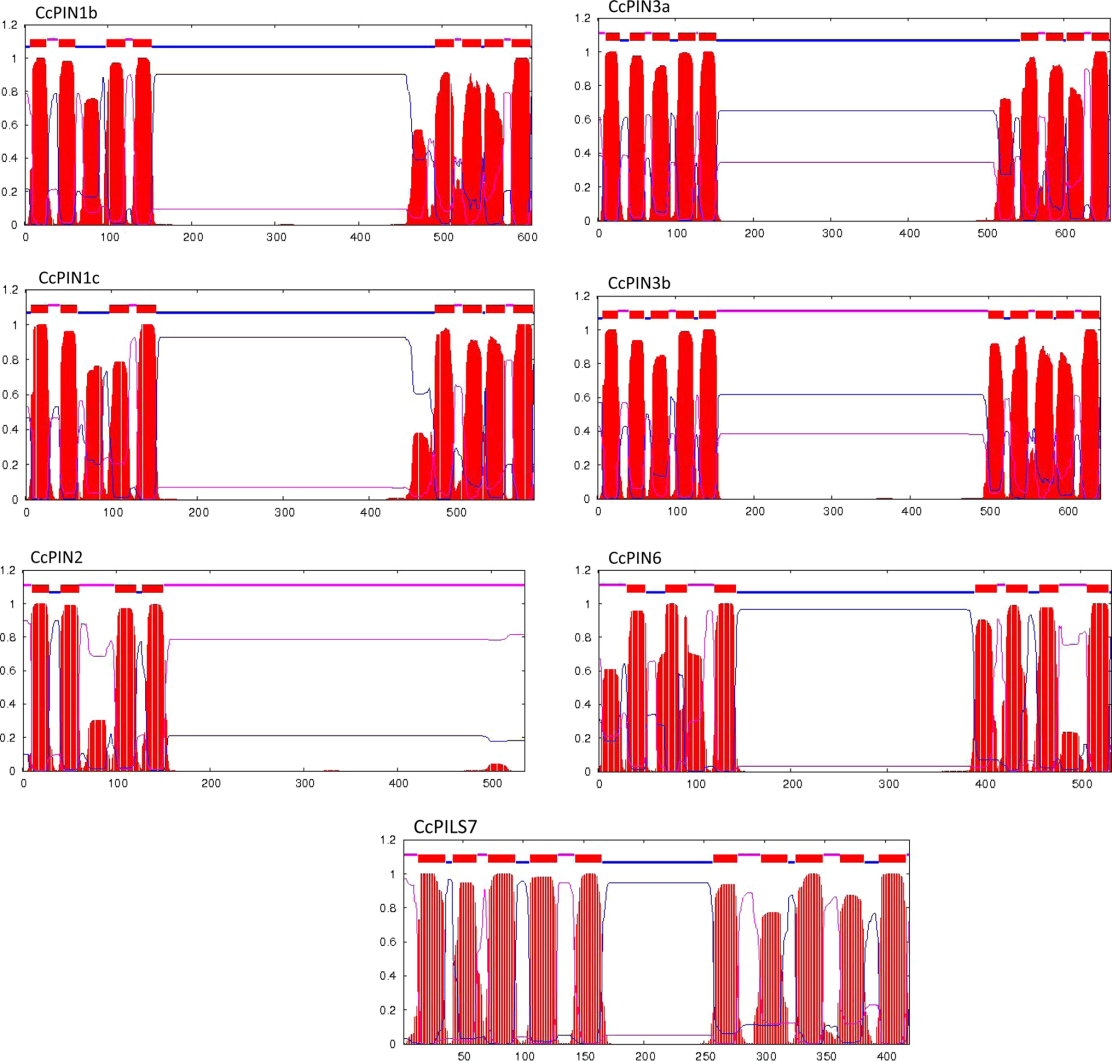
Prediction of the transmembrane regions for the selected hickory PIN-proteins through TMHMM2 software (trans~membrane prediction using Hidden Markov Models). The predicted transmembrane is shown in red, the regions predicted to be on the cytoplasmic side are marked in blue, and regions found outside are marked as pink color

Typical PIN-proteins have five highly conserved N- and C-terminal transmembrane helices and a less conserved middle hydrophilic loop of variable length (Ganguly et al. 2012; Bennett 2015). Based on the length of the middle hydrophilic loop, the Arabidopsis PIN-proteins were classified into canonical PINs with a longer loop (PIN1, PIN2, PIN3, PIN4, PIN7), or non-canonical PINs with an intermediate (PIN6) or shorter loop (PIN5, PIN8) (Bennett et al. 2014). The identified hickory PIN-proteins CcPIN1b, CcPIN1c, CcPIN3a, CcPIN3b exhibit the long hydrophilic loop (Fig. 3), while also in hickory, CcPIN6 has a loop of an intermediate size. In CcPIN2, the C-terminal transmembrane helices were completely absent (Fig. 3).

In plants, there also exists the family of PIN-LIKE proteins, which, in Arabidopsis, contain 7 members (PILS1–7) (Barbez et al. 2012). The PILS proteins are probably not directly involved in PAT but rather act at the endoplasmic reticulum where they are involved in regulating the intracellular auxin levels (Feraru et al. 2012). We identified one PILS gene in hickory, which we named CcPILS7 due to its positioning close to Arabidopsis PILS7 in the constructed phylogenetic tree (Fig. 2). CcPILS7 share topology with other PIN and PILS proteins, with five N- and C-terminal transmembrane domains. As in other PILS proteins, the N- and C-terminal transmembrane domains are connected by a short loop in CcPILS7 (Fig. 3).

**Fig. 4:**
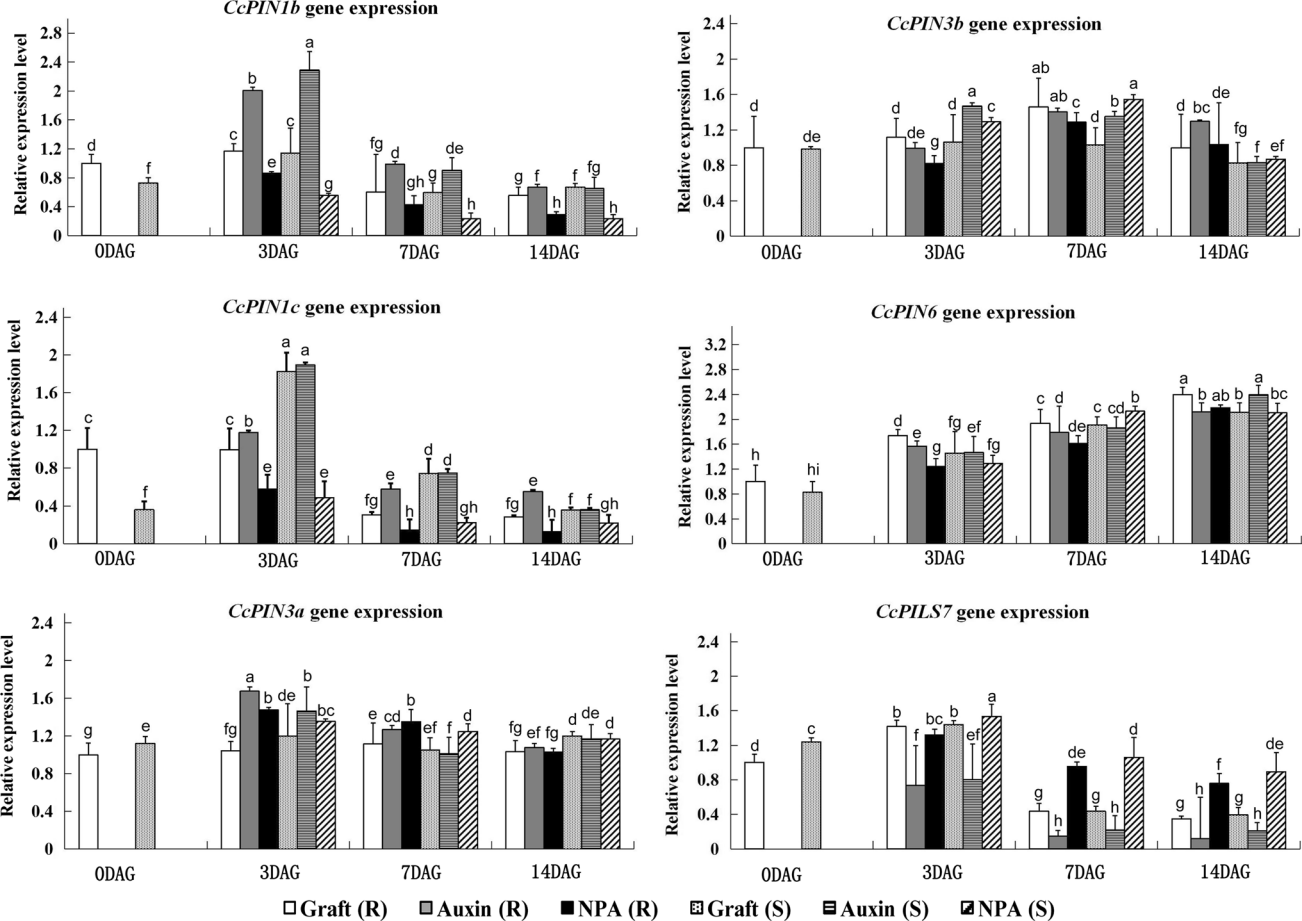
Expression analysis of hickory *PIN* and *PILS* genes during different time points of the grafting process (0, 3, 7, and 14 DAG). The transcript abundance was measured under control, and IAA- and NPA-applied conditions. Expression of the *CcPIN* and *CcPILS* genes was measured by qRT-PCR with actin as the reference gene. Error bars indicate the standard deviation of at least three different experiments. *R* rootstock, *S* scion. The relative significant difference between the samples is marked by small alphabet letters. Different letters indicate significant difference evaluated by Tukey post hoc test with *P* < 0.05. Sharing of same letter represents no significant difference

The expression during different time points (0, 3, 7, and 14 DAG) of the grafting process was analyzed for the hickory *PIN* genes. RNA was isolated from IAA- and NPA- and control conditions and expression levels were analyzed with RT-qPCR. The expression pattern varied for the individual *PIN* genes (Fig. 4). *CcPIN1b* and *CcPIN1c* showed similar patterns. The expression pattern of both genes reached a peak at 3 DAG in both root stock and scion. Application of IAA increased the expression of *CcPIN1b* and *CcPIN1c*, while NPA lowered the expression of both genes. The levels of *CcPIN6* mRNA increased throughout the test period, but neither IAA nor NPA seemed to influence the expression levels of this PIN gene. *CcPIN3a* and *CcPIN3b* were also expressed in all samples, but their mRNA levels did not change during the grafting process. The expression of *CcPIN2* was not observed during the grafting process. The expression for the *CcPILS7* increased at 3 DAG in control conditions. However, in the case of *CcPILS7,* IAA decreased the transcript levels.

**Fig. 5:**
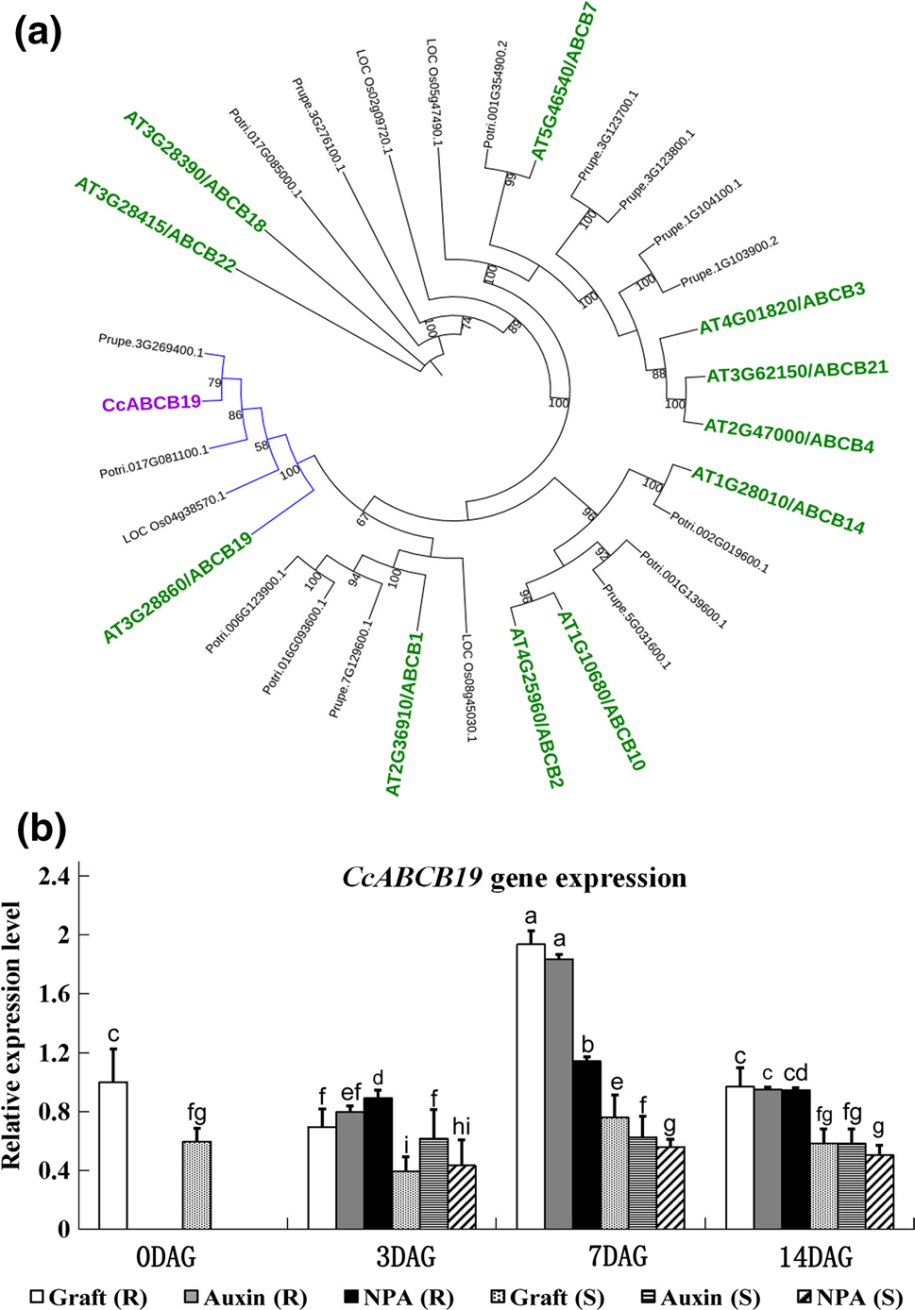
**a** Cladogram of CcABCB19 with ABCB family members from *A. thaliana*, *O. sativa*, and *P. trichocarpa*. Bootstrap values over 50 are shown. **b** Analysis of the relative expression level of the putative auxin-transporter *CcABCB19* at different time points during grafting (0, 3, 7, and 14 DAG). The samples were analyzed under IAA- and NPA- applied conditions. *R* rootstock, *S* scion. Different letters indicate significant difference at *P* < 0.05, calculated by Turkey’s post hoc test. Same letter represents no significant difference

The Arabidopsis gene *ABCB19* encodes an ABC transporter involved in polar auxin transport (Lin and Wang 2005). We identified the corresponding gene in hickory, and investigated its expression pattern during grafting. Phylogenetic analysis of the CcABC19 shows that it clustered with ABCB19 from Arabidopsis, and with similar proteins from poplar, peach, and rice (Fig. 5a). The expression profile for the *CcABCB19* gene was different between the rootstock and scion samples (Fig. 5b). In the rootstock, the expression of *CcABCB19* reached a peak level at 7 DAG. IAA treatment did not alter the expression levels, compared to the untreated root stocks. On the other hand, NPA application kept the *CcABCB19* expression at a lower level. Compared to root stock, the expression in scion was lower at all time points. Furthermore, in scion, there was no difference in mRNA levels at the analyzed time points.

### Identification and expression analysis of auxin influx transporters during grafting

**Fig. 6:**
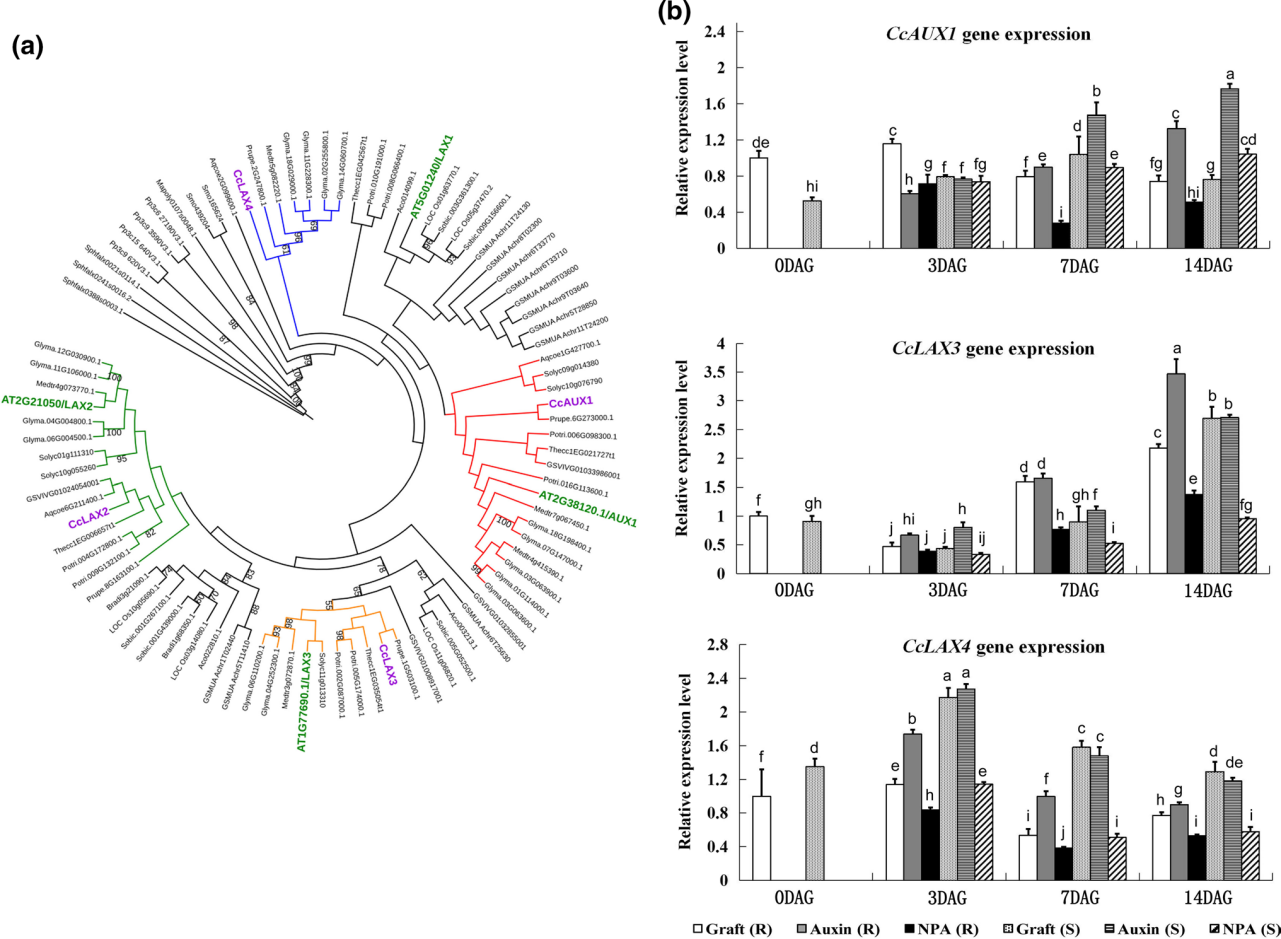
**a** Cladogram of the hickory CcAUX/LAX proteins with AUX/LAX protein family members from *A. thaliana* (AT), *V. vinifera* (GSVIV), *Solanum lycopersicum* (Solyc), *Musa acuminate* (GSMUA), *Brachypodium distachyon* (Bradi), *M. polymorpha* (Mapoly), *P. patens* (Pp), *S. moellendorfii* (Smo), *Sphagnum fallax* (Sphfalx), *T. cacao* (Thecc), *P. persica* (Prupe), *G. max* (Glyma), *M. truncatula* (Medtr), *P. trichocarpa* (Potri), *S. bicolor* (Sobic), *A. comosus* (Aco), *O. sativa* (LOC Os), and *A. coerulea* (Aqcoe). For orientation, the names of the hickory proteins are shown in purple, and the Arabidopsis proteins are in green. Clusters corresponding to different AUX/LAX proteins are shown in different colours. Bootstrap values over 50 are shown. The sequence identifiers are from Phytozome v12 (https://phytozome.jgi.doe.gov). **b** Relative expression analysis of the *CcAUX*/*LAX* genes at different time points during grafting (0, 3, 7, and 14 DAG). The samples were analyzed under IAA- and NPA-applied conditions. *R* rootstock, *S* scion. Different lowercase letters represent significant difference with respect to other sample at *P* < 0.05 analyzed by Tukey’s post hoc test

In Arabidopsis, the major auxin influx carrier is the AUX/LAX family represented by four highly conserved genes called *AUX1*, *LAX1*, *LAX2*, and *LAX3.* Using Arabidopsis *AUX/LAX* genes as query, we identified four homologous hickory genes. The phylogenetic analysis shows that Arabidopsis AUX/LAX1, LAX2, and LAX3 proteins locate to separate clades in the tree. Three of the hickory AUX/LAX proteins clustered with corresponding AUX/LAX proteins from Arabidopsis. These hickory proteins were named AUX1, LAX2, and LAX3 based on their positioning in the tree (Fig. 6a). The fourth hickory AUX/LAX protein was not clustering clearly with the Arabidopsis representatives of this protein family. Therefore, this protein was named as CcLAX4. In addition, in the case of the AUX/LAX family, in the phylogenetic tree, the proteins from non-seed plants were separated from the proteins of angiosperms.

The expression of *CcLAX3* is increasing throughout the experiment and reaches its peak at 14 DAG. IAA application increases the expression, at least at some of the investigated time points, while NPA represses the expression in comparison with the untreated sample. The level of *CcLAX3* expression is similar in root stock and scion. In addition, in the case of *CcLAX4*, it is clear that NPA treatment results in lower gene expression. The expression of *CcAUX1* was rather stable at the different time points of the analysis, and it was not clearly affected by the applications of IAA or NPA (Fig. 6b).

## Discussion

Successful grafting requires that both xylem and phloem are connected between root stock and scion. In the previous reports, we investigated the transcriptomic and proteomic changes in scion and root stock during grafting (Zheng et al. 2010; Qiu et al. 2016; Xu et al. 2017). In this study, we have demonstrated that IAA treatment improves the grafting success in Chinese hickory. PAT allows for uneven distribution of auxin in plant tissues. This asymmetric accumulation of auxin is extremely important for functional plant development, and it is also probably required for efficient regeneration and reconnection of vascular tissues during grafting (Melnyk et al. 2015). Important players in PAT are the proteins that act as influx and efflux carriers for auxin, such as the protein families AUX/LAX, PIN, and ABCB. In the current paper, we identified hickory genes encoding proteins in these families and investigated their expression during grafting after treatment with IAA or NPA.

Six *PIN* genes were identified in hickory. Genome analysis of many plant species has revealed that the *PIN* complement is relatively consistent; as eight different PIN protein types are reliably found throughout the angiosperms (Bennett et al. 2014). PIN-proteins share a secondary protein structure with five transmembrane helices at the N- and C-termini, coupled by an intracellular hydrophilic loop domain of variable length. One of the *PIN* genes we identified in hickory, *CcPIN2,* is lacking the C-terminal transmembrane helices. Furthermore, its expression was not detected here. This suggests that *CcPIN2* might be a pseudogene. The majority of PIN-proteins from liverworts to angiosperms also share a conserved long loop (> 150 amino acids) domain between the N- and C-transmembrane helices. PIN-proteins having such long loops are referred to as canonical PINs, whether PIN-proteins with a shorter loop (usually 30–120 amino acids) that lack some conserved elements are referred to as the non-canonical PINs (Bennett et al. 2014). In Arabidopsis, PIN-proteins (PIN1–PIN4, PIN7) with long hydrophilic loops separating the transmembrane domains are plasma membrane localized and co-ordinate many developmental processes (Benjamins and Scheres 2008). These are sometimes referred to as the canonical PINs due to their high degree of primary structure conservation (Bennett et al. 2014). However, there are also three non-canonical PINs (PIN5, PIN6, and PIN8) with shorter hydrophilic loops in Arabidopsis (Paponov et al. 2005; Krecek et al. 2009), of which PIN6 has loop of an intermediate size. These PIN-proteins with shorter loops are believed to be localized to the endoplasmic reticulum (Adamowski and Friml 2015). It has been suggested that the non-canonical PIN-proteins function in auxin homeostasis within cells rather than being involved in the transport between cells. A similar function is auxin homeostasis which is also suggested for the proteins in the PILS family (Feraru et al. 2012).

Some of the *CcPIN*s showed an increased expression level during grafting. In particular, *CcPIN1b* has an interesting pattern where the expression is induced after the auxin treatment during when also the grafting has a higher rate of success. The expression of *CcPIN1b* is induced both in root stock and scion. Treatment with auxin efflux inhibitor NPA reduced the expression of *CcPIN1b* and also of *CcPIN1c*. These results indicate that PAT mediated by mainly *CcPIN1b* could be important for the regeneration of vascular tissues during grafting in hickory. Studies from other plants suggest that auxin transport mediated by PIN1 has a major role in vascular differentiation. In pea epicotyls, disruption of the existing vasculature by wounding induced broad *PIN1* expression that narrowed and localized to the wound, after which xylem differentiated and vascular tissues connected (Sauer et al. 2006). There are also in Arabidopsis gradual changes in PIN1 localization during the regenerative processes after stem wounding (Mazur et al. 2016). Other studies from Arabidopsis revealed that the connection of veins in the leaf is preceded by the appearance of *PIN1* expressing outgrowths that extend from one existing vein to another (Scarpella et al. 2006; Wenzel et al. 2007).

We speculate that the *CcPIN* genes, such as *CcPIN3a* and *CcPIN3b*, which showed an unchanged expression pattern through the grafting process, may play a minor role in the vascular development in the stem. This speculation draws support from Arabidopsis where *PIN3* is required for altering the direction of auxin fluxes during tropic responses, such as gravitropism and phototropism (Ding et al. 2011; Rakusová et al. 2016). In the case of *CcPIN6,* the expression showed a small but gradual increase throughout the grafting; however, the expression was not altered by IAA treatment or NPA treatment. This expression pattern suggests that CcPIN6 is also involved in other processes than vascular development. In Arabidopsis, PIN6 has a dual localization at the endoplasmic reticulum and the plasma membrane and is suggested to be involved in regulating auxin homeostasis. The function of *PIN6* seems particularly important during the development of lateral and adventitious roots, since in Arabidopsis, both *pin6* knock-out and *PIN6* overexpressor lines show such phenotypical defects (Simon et al. 2016). Possibly, also *CcPIN6* is involved in lateral and adventitious root formation, rather than in vascular development. However, at this early stage of our investigation of the *CcPIN*s, we can merely speculate about the details regarding their physiological functions.

PAT is also dependent on the cellular auxin influx, which, in Arabidopsis, is mediated by the influx carriers LAX1–3 and AUX1. The *AUX/LAX* genes show mostly non-redundant expression and may have subfunctionalized during evolution to facilitate auxin-related developmental programs in different plant organs and tissues (Swarup and Péret 2012; Chai et al. 2016). Anyway, there is little information available concerning the role of the auxin influx carriers during vascular tissue regeneration and grafting. In this study, we identified four genes encoding putative auxin influx carriers in hickory. In particular, one of these, *CcLAX3*, showed a significantly increased expression during grafting. Moreover, the expression was repressed after treatment with auxin transport inhibitor NPA, indicating that auxin is required for the observed upregulation of *CcLAX3* expression. These data suggest that auxin influx carriers, such as *CcLAX3*, are upregulated to mediate PAT in scion and root stock during grafting. Interestingly, *LAX3* is upregulated in the presence of auxin also in other plants, such as Arabidopsis and sorghum (Péret et al. 2012).

It was recently shown that nuclear genomes may be transferred through the graft junction (Fuentes et al. 2014). This suggests that grafting could lead to the development of new species through the combination of two genomes from different species. Such genetic hybridization, known as allopolyploidization, has been used frequently in the development of many modern crop plants, as polyploidization often gives superior agricultural properties. Seemingly, grafting could, therefore, be developed to an efficient tool for generating new improved crops. Organelle movements, such as that of mitochondria, through the graft junction were also detected in a recent study (Gurdon et al. 2016). This is a finding that opens for the possibility to use grafting also for the systematic transfer of organelles between graft-compatible species. Potentially, grafting could, therefore, for instance, be used to transfer cytoplasmic male sterility (CMS) to agricultural and horticultural plants lacking CMS (Bergman et al. 2000). In further studies, we may also investigate the transfer of genetic materials through the graft junction in Chinese hickory. Anyway, the observations from our and other studies highlight the importance of detailed investigations of the grafting process, and implicate an even more significant role for grafting in plant breeding and biotechnology in the future.

In the present study, we have demonstrated that application of auxin improves grafting efficiency in hickory. Furthermore, we have identified hickory genes encoding proteins involved in polar auxin transport and demonstrated that some of these genes, such as *CcPIN1b*, *CcPIN1c* and *CcLAX3*, are upregulated during grafting. Now, we aim to identify genes which are upregulated as a consequence of the polar auxin transport in the grafting areas. Identifying these genes will be highly important to learn more about the biological processes during grafting. In long term, this information will allow for positive developments in breeding and commercial cultivation of hickory and other valuable horticultural plants.

### *Author contribution statement*

R.M.S.K performed experiments, analyzed results and wrote research; G L.X performed grafting experiments; Y. H. W., X.D.B., Z. L., T.S.C., G.W.B. and Y.D.L performed experiments and analyzed results; Z.B.S. designed project, analyzed data and wrote research; J.E. analyzed data and wrote research.

## Electronic supplementary material

Below is the link to the electronic supplementary material.

Supplementary material 1 (DOCX 15 kb)

## Abbreviations

ABCB: ATP-binding cassette subfamily B
AUX/LAX: AUXIN/LIKE-AUX family of auxin transporters
DAG: Days after grafting
NPA: 1-*N*-Naphthylphthalamic acid
PAT: Polar auxin transport
PIN: PIN-FORMED auxin transport protein
