# Supplementary material for: Auxin enhances grafting success in Carya cathayensis (Chinese hickory)
Source: Planta. 2017 Dec 6;247(3):761–72. doi: 10.1007/s00425-017-2824-3 (PMC5809526; doi:10.1007/s00425-017-2824-3)
Supplement: Supplementary file 1 — Supplementary material 1 (DOCX 15 kb) [file 425_2017_2824_MOESM1_ESM.docx]

**Supplementary Table S1.** Primers used in this study.

| **Gene** | **Purpose** | **Product Size (bps)** | **Primer sequence** |
| --- | --- | --- | --- |
| *CcPIN1b* | qPCR | 151 | 5’-GGGCCTAATTCCGGTCTTTCA-3’ |
|  |  |  | 5’-TGGCGTTCTTACCATTCACCA-3’ |
| *CcPIN1c* | qPCR | 165 | 5’-TTATGGCCTCTCAGCTTCACG-3’ |
|  |  |  | 5’-CCCGTTGGTGAAAACATTCCC-3’ |
| *CcPIN2* | qPCR | 155 | 5’-GAATCCCACTCTTGAAGGCCA-3’ |
|  |  |  | 5’-AGTCTCAGGGAATTGCTCAGC-3’ |
| *CcPIN3a* | qPCR | 160 | 5’-TTGCCGATCAGCCTCCAAATC-3’ |
|  |  |  | 5’-GGGCCCGTACTCTCTTTCTCT-3’ |
| *CcPIN3b* | qPCR | 167 | 5’-GATCTAGGCGCCAAGGAGATC-3’ |
|  |  |  | 5’-GGGCCACCTTCTTTCTCTCTC-3’ |
| *CcPIN6* | qPCR | 157 | 5’-CTCGTCGTGGTTGGAAGGAAG-3’ |
|  |  |  | 5’-CCATCCCAAGACCTGCATCTG-3’ |
| *CcPILS7* | qPCR | 155 | 5’-CTTGGCTTTCTCCCGTCAGAC-3’ |
|  |  |  | 5’-GCTGCAACCACGTATGTCCAT-3’ |
| *CcABCB19* | qPCR | 173 | 5’-CAGAACACGTTGAAGCTTGGG -3’ |
|  |  |  | 5’- GTGAATGCCTTCCCTCCATCA -3’ |
| *CcAUX1* | qPCR | 175 | 5’-CTGTATACTTTCGGCGGGCAC-3’ |
|  |  |  | 5’-CGTTGGCATGGGTCAGAAGTT-3’ |
| *CcLAX2* | qPCR | 156 | 5’-GGGTAGGGGCTTACACGATCA-3’ |
|  |  |  | 5’-GTAGAGGTGGAGGAGGGCATT-3’ |
| *CcLAX3* | qPCR | 166 | 5’-GTTTGGATTTGCTTGCACCCC-3’ |
|  |  |  | 5’-AACAGTTGAGTTGATGGGGCC-3’ |
| *CcLAX4* | qPCR | 155 | 5’-TGGAATCACAGAGGCCTTTGC-3’ |
|  |  |  | 5’-CTCCATGTCCGTTTCCTGTGG-3’ |
| *Actin* | qPCR Reference  gene | 176 | 5’-GCTGAACGGGAAATTGTC-3’ |
|  |  |  | 5’-AGAGATGGCTGGAAGAGG-3’ |
| *CcPIN1b* | Full gene cloning | 1,779 | 5’-ATGGGTTTCTGGTCACTCTTTGAG-3’ |
|  |  |  | 5’-TCAAGACAAGATCCACATGTAGAC-3’ |
| *CcPILS7* | Full gene cloning | 1263 | 5’-ATGGGTTTCTGGTCACTCTTTGAGGTG-3’ |
|  |  |  | 5’-TCAAGACAAGATCCACATGTAGAC-3’ |
| *CcLAX3* | Full gene cloning | 1398 | 5’-ATGGCTTCTGAGAAGGTTGAGACTG-3’ |
|  |  |  | 5’-TCAAGCCTTGTGTGGGGGACACTGG-3’ |

**Supplementary Table S2.** The *C. cathayensis* genes investigated in this study.

| **Gene name** | **ORF length (bp)** | **Protein length (aa)** | **MW (kD).** | **pI** |
| --- | --- | --- | --- | --- |
| *CcPIN1b* | 1779 | 592 | 65.94 | 8.81 |
| *CcPIN1c* | 1824 | 608 | 63.88 | 8.95 |
| *CcPIN2* | 1611 | 536 | 58.69 | 9.09 |
| *CcPIN3a* | 1983 | 660 | 72.04 | 7.69 |
| *CcPIN3b* | 1932 | 643 | 70.06 | 8.45 |
| *CcPIN6* | 1599 | 532 | 58.04 | 9.28 |
| *CcPILS7* | 1263 | 420 | 46.22 | 5.38 |
| *CcABCB19* | 3639 | 1212 | 131.54 | 8.79 |
| *CcAUX1* | 1425 | 474 | 53.48 | 8.29 |
| *CcLAX2* | 720 | 239 | 26.72 | 9.28 |
| *CcLAX3* | 1398 | 465 | 52.56 | 8.86 |
| *CcLAX4* | 1299 | 432 | 48.85 | 5.24 |
